# Current News

**Published:** 1901-05

**Authors:** 


### Current News.

### FOREIGN RELATIONS COMMITTEE OF THE NATIONAL ASSOCIATION OF DENTAL FACULTIES.

THE Foreign Relations Committee, to whose jurisdiction has been referred certain foreign educational matters affecting the interests of the National Association of Dental Faculties, has been in receipt of a great mass of correspondence concerning the status of the distinctive American dental degree in Europe, and the effect upon public opinion there of the almost open sale of diplomas issued by fraudulently conducted but regularly chartered schools in the State of Illinois, and their quasi-recognition by the Illinois State Board of Dental Examiners, who, it is charged, have admitted to the State dental examination and granted permits to practise to foreign possessors of these irregular degrees obtained, in some instances, after their having been in this country for less than a month.

At the last meeting of the National Association of Dental Faculties, upon the recommendation of the Foreign Relations Committee, the work of the suppression of the irregular and fraudulent schools was placed in the hands of the Committee on Law, but as the first-named committee is in constant communication with American and other dentists abroad, and with our own government and its officers, in relation to the condition of the American degree in foreign countries, it has been deemed best, at the suggestion of such correspondents, to furnish our dental journals with abstracts from a few of these communications, that the profession in America may be made aware of the consequences resulting from the vicious character of some of the Illinois legislation. The first instalment of this was furnished without comment to the principal journals for their April issues, and the following is in supplement to that. The committee is necessarily dependent upon our professional magazines for this courtesy, and it is glad to say that all have expressed a hearty willingness to aid in the good work to the extent of their power.

W. C. BARRETT,
*Chairman.*

(Copy.)

UNITED STATES CONSULATE,
MUNICH, February 25, 1901.

DR. W. C. BARRETT,

*Chairman Foreign Relations Committee, National Association Dental Colleges:*

ESTEEMED SIR,—I have the honor to acknowledge your letter of the 9th inst. The work undertaken by me is to bring me no other reward than the restoration to honor and acceptance everywhere of the American degree in dentistry, than which no other country merits it better.

The report of the committee you sent I had read ere I prepared my last despatch sent to the government at home and here, and in my recent despatch mailed last week to Washington I supplied evidence against one of the supposed *suppressed* institutions, the old "Academia Illinois;" the diploma dating from May 24, 1899, although obtained much later and issued by Dr. B. E. Winkler and Dr. B. I. R. Ungelenk. Of the diploma as well as the notary's certificate (which is *printed,* as you will see, so that the notary only had to write in his own name) I hand you photographic copy herewith for further use. The whole case will, of course, be duly reported to you from Washington, and I think will undeceive you as to all parties of the nefarious traffic being in jail.

You will oblige me if you will kindly state the names of all the parties thus far arrested, and promptly have me informed of any new one added to those jailed, as it is all-important that we have these names registered here.

Very respectfully,
(Signed) JAMES H. WORMAN,
*Consul.*

(Enclosures.)

1. Copy of diploma of Academia Illinois to Bosch. Signed, Dr. B. E. Winkler, Pres., Prof. Op. Dent.; B. I. R. Ungelenk, Sec., Prof. of Chem.
2. Kreuker's certificate as notary.
3. Copy of letter from the police here.

CONSULAR SERVICE, UNITED STATES OF AMERICA,
MUNICH, GERMANY, February 12, 1901.

DR. W. C. BARRETT,
208 Franklin Street, Buffalo, N. Y.:

DEAR SIR,—Just now a very peculiar case is up here, and I am anxious to have your assistance at once. Under this date I write to the State Board of Illinois as per enclosed, also to the Secretary of the Board of Health of Illinois, addressing him at Springfield and requesting two copies of the annual report or of the law as amended for examinations by the State board, to be certified to by the Secretary of State *and the German Consul.* This is all-important.

This Gumpoldt has *no* Roumanian degree, and was in the United States only a few weeks, possibly only a few days. I know when he went and returned. He has a diploma from Weil and two certificates from the State Examining Board, as my letter to Reid discloses, both referring in date and contents, Weil having been the interpreter. Our German Consul in Chicago has notified the courts here that the Illinois Board is competent to grant such certificates, and that the signatures of three make it a *bona fide* certificate. The law requires annual meeting for the granting of licenses. Members of the board can only grant temporary certificates, renewal necessary, and registration in County Clerk's office within six months, all of which was *not* done.

Yours truly,
(Signed) J. H. WORMAN,
*Consul.*

No. 48.

CONSULATE OF THE UNITED STATES OF AMERICA,
MUNICH, GERMANY, February 13, 1901.

HON. DAVID J. HILL,
*Assistant Secretary of State,* Washington, D. C.:

SIR,—Most respectfully referring to my despatch No. 38, under date of December 29, 1900, regarding the sale of dental degrees in Germany and to the preceding communications from this Consulate to your Department on the same subject, I have the honor to report further:

[The first part of this despatch of Consul Worman, of New York, consists of a review of what has been previously reported concerning the "Academia Illinois," of Chicago, and the "Wisconsin College of Dentistry," of Milwaukee, both of which have been suppressed, and of extracts from the report of the Foreign Relations Committee made at Omaha, July, 1899, in which the enormity of the foreign diploma traffic on the part of certain Chicago institutions was fully set forth. This is omitted as having been substantially published before.]

The German dental journals have exposed and denounced the swindling institutions fraudulently issuing diplomas, but so indifferent are the German authorities to protect the dignity of American degrees that it is difficult to enlist them for prosecution that will restore American dental honors to the distinction to which they are entitled.

In 1897 the *Journal für Zahnheilkunde,* XII. Jahrgang, No. 42, review a prospectus of the "Academia Illinois" which they had secured from Chicago, and thoroughly exposed its illegal business and the worthlessness of its diplomas. (A copy of this article and a translation are hereto annexed marked Exhibits J and K.)

(An exhibit to the same purpose marked L from the *Zahnarztliche Rundschau,* Berlin, July 15, 1900, page 6860, is also hereto annexed. A translation of the same is marked Exhibit L 1.)

To what low estate American university training and honors have fallen in Germany on account of this disgraceful traffic is perhaps better apparent from these technical journals than from the manner in which the subject is treated by the secular press, and even by the comic journals.

Can any American of spirit sit idly by and see such disgraceful caricatures launched against educational enterprises, and that with justification by reason of such want of supervision on the part of so great and cultivated a State as Illinois, whose chief city where this nefarious traffic centres has a reputation to lose as well as to make, second only to the metropolis of our great country?

(A cartoon from the *Lustige Blätter,* showing a penny-in-the-slot diploma machine, is hereto annexed, marked Exhibit M.)

This publicity has not always treated of the swindling institutions alone, but has very often generalized the whole subject, not only of American dental degrees, but all American educational honors, and has created in the minds even of educated Germans a prejudice against our institutions of learning which is as unfortunate as unjust, and is proving and will prove still more in the future detrimental to American practitioners in Germany, and probably lead ultimately to the exclusion of American degrees in South Germany, like in the north, where their holders are practically denied the right to use or practise under them. (See Exhibits K 1, K 2.)

The courts of North Germany have had until recently much more to do with this question than those of the Southland, and the manner in which they have commented upon our educational institutions, though largely due to want of understanding of the organization of these schools, has nevertheless reflected the general unfavorable opinions entertained here. Such expressions of eminent men could have but helped to strengthen and confirm the prejudices of those of lower estate.

*It is simply a question of time, and that not so far away, when the courts of the Southland will re-echo these same unjust sentiments, if the flood-tide of public opinion is not promptly stemmed by discriminate and effective legislation at home. It will not do for the legislators of the State that has brought such disgrace upon our common country and good name to plead impairment of interests, which have become incidentally vested under vicious legislation, as a reason why no more effective measures have prevailed against the nefarious traffic and its disgraceful results.*

The statement of Dr. W. C. Barrett, Chairman of the Foreign Relations Committee of the National Association of Dental Faculties (see Exhibit 1½), that thus far the National Association of Dental Faculties has failed to carry through such legislation as the conditions exact, is best supported by citation of court reports here.

A very recent expression from the criminal court of Breslau, although restricted to the "Academia Illinois," no doubt influenced a judgment rendered by the District Court of Nuremberg, and caused a most indiscriminate and wholly unwarranted denunciation of the "Chicago College of Dental Surgery," one of the most reputable of American schools. (A copy of this decision and translation thereof are hereto annexed marked Exhibits M 1 and M 2.)

The judgment in Breslau was given in July, 1900, and probably the charter of the "Academia Illinois" had been revoked, but the court did not seem to care to discriminate between extinct and existing institutions.

An English rendering of the decision is as follows: The professors of the "Academia Illinois" were tradespeople, policemen, etc., who did a flourishing business by trafficking in diplomas in all professions, and sent their swindling advertisements to dentists abroad, unfortunately not always without success. One of the deans of these institutions was one Abert, sentenced to the penitentiary in Breslau, escaped to America, and was there forced to undergo a prolonged treatment in a penitential "water cure."

What the Committee on Foreign Relations of the National Association of Dental Faculties said as far back as 1898, at Omaha, surely goes to the root of the matter.

Their report expressed the earnest hope "that as soon as the professional men of the State of Illinois are aroused from their lethargy and made to comprehend the enormity of the conditions, they will present the matter before the Legislature in its proper light, and the disgraceful law will be so amended that it will not apply to educational institutions, and the charters already issued under it will be very promptly cancelled."

It is evident, from what has been heretofore said, that this good work has not yet been accomplished.

As here truly said, however, the remedy lies at home, and I hope at a very early day to most respectfully submit some suggestions that will be practical as to procedure in Illinois, obtained as the result of the criminal trials and investigations by the Attorney-General's office here; and perhaps also regarding diplomatic representations that seem to be called for to put an end to the prejudicial part taken in some of the cases by the Imperial German Consul in Chicago, or his subordinates in his name.

I have the honor to be, sir, your obedient servant,

(Signed) James H. Worman,
*United States Consul.*

Exhibit.

(Translation.)

Munich, December 17, 1900.

To the Chief of Police:

*In the matter of Franz Xaver Bosch, for using here the title of Doctor of Dental Surgery.*

In reply to your letter of the 24th ultimo, I beg leave to state that the "Academia Illinois" still has the right to issue dental diplomas, but is known to have abused such privileges, and will not be permitted to exercise the privilege much longer.

The issuing of such diplomas is only permitted when the applicant has pursued a regular course of study of several years, and has passed a formal examination.

Even under the most favorable circumstances, a thoroughly qualified candidate would not be admitted to examination at one of our reputable dental colleges unless he had pursued his studies there for at least six months. According to the new regulations, the candidate is admitted to examination only after eighteen months of study in the college where he wishes to graduate.

The institution in question is said to have issued diplomas to applicants on payment of a certain sum of money, who have not complied with these preliminary requirements, as has been proved at the trial of Emil Gumpoldt, in the Criminal Court No. 2, at Munich, on December 10, 1900.

The holders of diplomas like the one issued to Bosch cannot even practise in America.

Such diplomas are mainly used abroad to deceive a public unable to discriminate between such and those approved by the United States Commissioner of Education.

The American government is assiduously striving to suppress these spurious institutions.

There are only two still in existence,—viz.:

1. The Cosmopolitan Post-Graduate College of Dental Surgery at Chicago.

2. The German-American Dental College, also at Chicago.

I, therefore, respectfully request you to have the diploma in question confiscated and transmitted to me, and vouch for its return. I desire to send it to the United States Commissioner of Education, in order to furnish official evidence that such a spurious diploma was issued by the said institution. The Commissioner of Education would then be in a position to pronounce on the illegality of the diploma, and to take steps to suppress the institution.

The anticipated reply of the State Department will be given without delay, since I have already called its attention to these swindling institutions, and have had not only its approbation of my action in the matter, but have been instructed to co-operate in the settlement of this affair with the government here.

I have the honor to be, sir, your obedient servant,

(Signed) JAMES H. WORMAN,

*United States Consul.*

(Here follows as "Exhibit I" a copy of an article from the *Dental Cosmos* of July, 1900, page 700 of vol. xlii., under the title, "A Slump in the Diploma Traffic.")

EXHIBIT.

(Translation.)

(From the *Journal für Zahnheilkunde*, No. 42, 1897.)

A CHAPTER ABOUT DOCTORS OF DENTAL SURGERY.

For some time past eulogies of the latest swindling institution, the "Academia Illinois," have been circulating in Germany.

To-day accident has thrown into our hands a prospectus of this "institute." The swindling intent is so apparent in this circular that he must, indeed, be dumb who could fall into its hands.

At the same time we have received information from Chicago that the postmaster there has been instructed not to deliver letters and money remittances addressed to the "Academia Illinois," but to send them to the Dead Letter Office in Washington, to be returned to the writers and senders, as according to American law the mails cannot be used for fraudulent purposes.

To avoid this prohibition, this worthy crowd has pasted over the address of the "Academia Illinois" on the circular and given the address of a boarding-house lodger who has no actual residence there, to enable them to continue the swindle and enhance the difficulties of the prosecution.

In order to escape the effect of the law of April 7, 1897, regarding the necessity in Prussia of ministerial permission to use a foreign doctor's degree, the president, whose signature appears on the circular, has the audacity to assume that this "rag" which he calls a diploma would be acknowledged in Prussia. (He calls himself in the circular "Magnificenz" and "Rector Magnificus.")

We have, on the part of our society, sent properly certified copies of this circular, as well as the information received from Chicago about this swindling concern, to all proper officials, to place a bar to the use of this diploma which the notarially certified documents from Chicago cannot and will not break through.

In the circular the president calls himself "Magnificenz," and names among the "Founders" four victims, three in Germany and one in Brussels. We hope that these pilloried gentlemen will soon find out that they themselves have been swindled, and will not lend themselves to the stool-pigeon service which is attributed to them in the circular.

(From the *Neusten Nachrichten*, Munich, February 6, 1901.)

THE RIGHT TO THE TITLE "AMERICAN DENTIST."

The dentist Emil Gumpoldt, who acquired the title of "American Dentist" in America, advertised as such in several newspapers, and had a sign made bearing that title. December 10 of last year Gumpoldt had to appear in court to account for the title which he had no right to claim, and was sentenced to five days' arrest or a fine of twelve dollars and fifty cents.

The court of justice has taken the matter in hand, and the American Consul, Mr. J. H. Worman, was present at the transaction, as witness and competent judge. Mr. Worman asserts that the certificate which Gumpoldt received (from the Illinois State Board of Dental Examiners), and which he claims entitles him to "American Dentist," is not genuine, as it should be signed by a board of examiners of five members. Gumpoldt's certificate shows but three signatures, and is therefore only provisory, or not valid. If the certificate were genuine and signed by the full number of censors, it would pass for a "license of trade," and only entitle the holder to practise in the State of Illinois (where it was issued). The certificate bears the signature of the German Consul at Chicago,—not as a competent judge, however, but to confirm that the wording of the German copy is genuine. A diploma such as Gumpoldt pretends to possess Mr. Worman considers a fraud of individuals whose principal aim is to rob foreigners, and Gumpoldt claims to have become their victim, and his case should serve as a precedent to his colleagues.

The Consul states that such diplomas cannot be granted by law unless the student has lived in America a certain length of time and has passed through a course of studies. Mr. Worman considers it his duty to oppose such frauds, and has taken steps to insure the punishment of such swindlers by his government.

The testimony taken showed that Gumpoldt arrived in the city of Chicago in the spring of 1900, obtained a diploma and the degree of Doctor of Dental Surgery from the "Cosmopolitan Post-Graduate Medical College," was admitted to and passed the examination of the "Illinois State Board of Dental Examiners," received a license to practise in the State of Illinois, and returned to Germany, leaving Munich after the middle of April and arriving back again early in June of the same year, having been gone but little more than four weeks. He had never previously pursued any course of dental study.

EXHIBIT.

(From the *Zahnarztliche Rundschau,* Berlin, July 15, 1900.)

AMERICAN DOCTOR'S DIPLOMAS.

BY DR. A. E. MILLER, OF CHICAGO.

The European and more especially the German Dental press has within the last few years taken up the question of American diplomas, and so much has been written about it, without giving any accurate statement of the conditions prevailing in America, that I am prompted to give an impartial elucidation of American college institutions.

The writer, after graduating from a high school, attended the universities of Berlin and Leipzig, and was, as student and teacher, connected with colleges here, and may, therefore, be considered a competent judge.

The so-called dental colleges, dental schools, etc., are, as a rule, private enterprises, and, according to the will of the Legislature, are not founded in the interest of science and humanity without yielding pecuniary profit to the managers. The words, "incorporated under the laws of the State," do not mean that the institutions are State institutions, but that they have been founded like insurance companies, large hardware concerns, liquor enterprises, etc.

Therefore, the doctor's degrees of the college here are not to be confounded with the state diplomas in Germany, but would rank with the apprentice certificates of any of the above-mentioned business concerns. There is this difference, however: while the State in the exercise of the police power does not concern itself as to whether apprentice certificates of hardware concerns are based on truth, and does not vouch for the qualification of their holders, it manifests an interest in those young people who have received a doctor's diploma and are desirous of practising dentistry, to see that their diplomas do practically guarantee the qualification of the holders. Therefore there is in every State a "State Board of Dental Examiners," whose duty it is, in the interest of public health, to see that no unqualified persons are admitted to practise dentistry. This is carried out by subjecting all candidates to an examination, no matter whether they have attended a college or not. From this examination are only exempt such as are in possession of a doctor's diploma from institutions whose reputation warrants a thorough qualification of the respective holders of diplomas and who have obtained their licenses to pursue the practice of dentistry by virtue of their diplomas.

IMPERIAL GERMAN CONSULATE,
CHICAGO, June 28, 1899.

In regard to your letter of the 11th of this year, I write to state that the Cosmopolitan Post-Graduate College is permitted by law to give the degree of Doctor of Dental Surgery after passing examinations. The course lasts from eleven to thirteen weeks, and is not arranged in classes. The directors of the college, Dr. Weil and others, have a good reputation so far as I know.

If the degree given in this college has the same value as those given in other colleges, I am not prepared to say. I have neither means nor opportunities to investigate the many institutions which exist in this country and which are not under our control.

Concerning the value of American doctor diplomas in Germany, you can best be informed in Berlin.

The Imperial Consul,
(Signed) F. A. LETTENBAM.

(From the *Basel Vorwärts* of March 12, 1901.)

The Doctor Factory in Philadelphia, which, as is well known, on the payment of several hundred dollars, helps every one to a Doctor Title, is now so crowded with work that, to furnish the paper for doctor diplomas, eight large paper-factories are working day and night uninterruptedly. And even then thousands of aspirants for the doctor title must be turned away daily or put off to future years. Among the applicants for the coveted title are numerous ladies; the modern American lady knows no higher ambition (or has no higher pride) than to place Dr. before her name.

DRESDEN, January 30, 1901.

PROF. DR. W. C. BARRETT:

HONORED PROFESSOR,—The Society of Graduate Dentists in Dresden sends you a copy of one of the Cosmopolitan Post-Graduate College diplomas. With this diploma they receive the title of doctor (D.D.S.). The Society of Post-Graduate Dentists in Dresden consider this title worthless. We beg, dear doctor, that you explain to the German Consulate at Chicago the value of this college, that they may send us an official statement of its worthlessness as speedily as possible, and that we may deal justly with the Consulate. The Foreign Relations Committee has already been notified.

In one of your letters to Dr. Miller, of Berlin, were some very interesting facts about the Cosmopolitan College. We hope it is possible for you to obtain the official statement for us, and that the college, which we have no doubt has been closed by the law, and the director of the college shall meet with their reward.

It will then be an easy matter to persuade the Ministerium in Saxony of the worthlessness of the college and to obtain the punishment of these gentlemen.

Believe me, with very great respect, and the compliments of the Society of Graduate Dentists of Dresden,

(Signed) BOENHERR.

(Translation.)

DRESDEN, January 14, 1901.

DR. W. C. BARRETT:

DEAR PROFESSOR,—Your kind letter and the report of the Foreign Relations Committee were received a short time ago. Allow me to thank you most sincerely for having sent the explicit account to our colleague, Dr. Boenherr. Our association has carefully weighed and considered the letter, and is confident we are now able to take a further step in doing away with the diploma swindlers in Germany, as well as to unmask the bearers of the false title of "Cosmopolitan Post-Graduate College of Chicago."

It is difficult to understand how it was possible that the "Ministerium" should acknowledge the title after the very superficial examination, and, moreover, how it could be permitted that the bearer of this diploma could make use of the same. Thanks for the excellent proof that we now have, owing to our colleagues. We sincerely hope that we shall be able to convince the officials of the falseness of the title, and thereby do away with this swindle, especially since it is of vital interest to the whole dental world.

As soon as this matter is settled we shall not fail to send you and all our colleagues in America an accurate report. So far we have not secured the official certificate of the German Imperial Consulate of Chicago, by aid of which we can readily overcome all difficulties. Although we made known to the Consulate of Chicago about nine months ago the facts concerning the existing condition of the "Cosmopolitan College," we received an evasive rather than an explanatory answer. This made us consider the matter seriously, if not suspiciously; in fact, to such an extent that we may report the deficiency to the "Chancellor's" office in Berlin.

It seems impossible that the Consulate should be ignorant of the worthlessness of the said college, when you, as well as Dr. Brophy, Dr. Kirk, and Professors Miller and Hesse, of Germany, have given us such definite information. We have also reports from the following sources: *Dental Cosmos,* March, 1899, page 287; *Items of Interest,* No. 7, July, 1899. Can you in any way explain why the German Consulate of Chicago gave us such unsatisfactory answers?

You would confer a great favor on us by giving us an official document about the "School of the Cosmopolitan Post-Graduate College." Of course, we shall pay all expenses in connection with this.

Sincerely yours,

(Signed) C. Blochman,

*Society of Graduate Dentists of Dresden.*

To W. C. Barrett,

Chicago:

Dear Professor,—Your kindness requires especial thanks. I hope that your explicit document will make an impression upon the ministerial offices.

It may be of interest to you to know that the Saxon Minister received a favorable report from the German Consul regarding the questionable college, and said Consul at Chicago is therefore not free from complicity. We must therefore be under great obligations if you could obtain a proper document from the general Consul in Chicago, telling us of the quality of said college.

Yours,

(Signed) Yoenken, D.D.S.

Exhibit.

Munich, January 5, 1901.

Dr. J. G. Reid,

Chicago, Ill.:

Esteemed Sir,—I am informed that you are president of the Illinois State Board of Dental Examiners.

(1) Will you, as such, kindly inform me whether candidates for the practice of dentistry in your State are not obliged to have pursued a course of study, for at least a short period, in some school of dentistry before being admitted to examination before your board?

(2) Kindly reply how it comes that certificates are issuing with Dr. Smyser as secretary and Dr. Jocelyn as president of your Board of Examiners?

(3) Are certificates complete and legal if issued without the signature of yourself or of Dr. Pitner, if issued September, 1900?

An early reply is most respectfully requested.

Very truly yours,

(Signed) JAMES H. WORMAN,
*United States Consul.*

EXHIBIT.

STATE BOARD OF DENTAL EXAMINERS,
CHICAGO, January 22, 1901.

JAMES H. WORMAN,
*United States Consul,* Munich, Germany:

HONORED SIR,—Your letter of January 5 addressed to me as President of the Illinois State Board of Dental Examiners was duly received. While I am not the president of the board, I am its secretary, and such correspondence is usually delegated to such officer to answer.

(1) Candidates for examination do not have to present credentials of any kind to be admitted before the board. Any one who may desire can come before the board irrespective of how much or how little knowledge he may possess on dental subjects. The board determines his qualifications after the examination.

(2) Licenses bearing the signatures of only three members of the board can only be explained on the ground that the former secretary did not send the license to Dr. Pitner or myself for signing, and the majority of licenses abroad would not have been signed by us any way, especially those issued during the past two years.

(3) To my knowledge the courts of this State have never passed upon the question of the requisite number of names that shall appear on the document to make it legal. From what we see, one is led to suppose that three names are sufficient to make it legal. The law says in plain terms that the license shall be signed by the members thereof. So long as I remain secretary of the board no license will appear in your country without the required number of names.

There are times when the board might be vacant for some time. There are times when a member of the board could not be reached, hence his signature could not be obtained, but these are only exceptional cases, and not general.

I enclose you with this letter a copy of the law, that you may familiarize yourself.

Any further information you may desire I will gladly furnish if within my power.

Very respectfully yours,

(Signed) J. G. Reid, D.D.S.,
*Secretary.*

United States Consulate,
Munich, Germany, February 7, 1901.

Dr. J. G. Reid,
*Secretary Illinois State Board of Dental Examiners,* 1006 Champlain Building, Chicago, Ill., U. S. A.:

Dear Sir,—Please accept my thanks for your prompt answer and your kind explanations, as well as for the enclosure with your favor of 22d January. Kindly favor me with two new copies of same. May I now hope to have your answer to the following queries:

(1) Why was one Emil Gumpoldt granted a license to practise dentistry in Illinois under date of May 5, 1900?

(2) Why was the paper signed by only three members, and why was a new issue of this certificate made in September?

(3) What evidence of fitness did he furnish for such licensing, and why do the two certificates differ in contents?

(4) Have these certificates any value so long as they were not filed in the County Clerk's office within six months?

(5) How came it that Dr. Weil, himself interested in "graduating" students in dentistry and the head of a suspected institution and excluded from the list of reputable colleges, was permitted to conduct the examination of said Gumpoldt, or at least to assist as an interpreter?

(6) Are the examination papers of this candidate on file at your office, also his diploma?

Kindly give me answer by return post and oblige,

Yours truly,

(Signed) J. H. Worman.

### AMERICAN MEDICAL ASSOCIATION, SECTION ON STOMATOLOGY.

The next meeting of the American Medical Association will be held at St. Paul, Minn., June 4, 5, 6, and 7, 1901. The Section on Stomatology presents the following programme:

Chairman's Address. R. R. Andrews, Cambridge, Mass.

### SYMPOSIUM ON STATE BOARDS OF DENTAL EXAMINERS IN THEIR RELATION TO THE PROFESSION AND THE COLLEGES.

1. Methods of Appointment: (*a*) By State Universities, New York; (*b*) By State Boards of State Officials, *ex-officio,* Nebraska; (*c*) By Governors on Recommendation of the Profession. William Carr, New York City.

2. Revenue for conducting the Work of the Boards of Examiners: (*a*) By Taxation of the People; (*b*) By Fees from Examination of Candidates; (*c*) By Taxation of the Profession. George L. Parmele, Hartford, Conn.; V. E. Turner, Raleigh, N. C.

3. The Dental College Standard: (*a*) Is it what it should be? (*b*) If not, what Improvements should be made? (*c*) How may the Requirements be improved? Charles Chittenden, Madison, Wis.

4. Licensing: (*a*) By Examination; (*b*) By Diploma. J. A. Libby, Pittsburg, Pa.

### SYMPOSIUM ON DEGENERACY OF THE PULP.

1. Preliminary Work. Eugene S. Talbot, Chicago.

2. Literature of the Pulp. Vida A. Latham, Roger's Park, Ill.

3. Cutting, Staining, and Mounting. Martha Anderson, Moline, Ill.

1. Local Anæsthesia. A. H. Peck, Chicago, Ill.

2. A Remedy for Certain Injustice both to the Insured and the Company. W. E. Walker, Pass Christian, Miss.

3. Periods of Stress and their Dental Marks. Jas. G. Kiernan, Chicago.

4. Surgical Treatment of Cleft Palate. G. V. I. Brown, Milwaukee, Wis.

5. Persistent Neuralgia after Root Filling. E. K. Wedelstaedt, St. Paul, Minn.

6. Infectious Diseases. Alice Steeves, Chicago.

7. Simple Gingivitis. Geo. T. Carpenter, Chicago.

8. Military Dental Practice: Its Modifications and Limitations. Henry D. Hatch, New York City.

9. The Tongue as a Breeding-Place for Bacteria. M. H. Fletcher, Cincinnati, Ohio.

10. Pathology of the Alveolar Process. Eugene S. Talbot, Chicago.

11. Tuberculosis of the Alveolar Process and Surrounding Tissues and a Few Methods of Differential Diagnosis. V. A. Gudex, Milwaukee, Wis.

The officers of the Section invite all to be present and take part in the discussions.

Those who desire to join the Association must obtain credentials from their State or local dental societies and pay five dollars to the secretary of the Association. This will entitle them to the Journal for one year.

R. R. Andrews,
*Chairman Section on Stomatology.*
Eugene S. Talbot,
*Secretary Section on Stomatology.*

---

### NOTICE.

War Department, Surgeon-General's Office.

Candidates for appointment as Dental Surgeons in the United States army will be examined in the following-named branches: Anatomy, Physiology, Histology, Physics, Chemistry, Metallurgy, Dental Anatomy and Physiology, Dental Materia Medica and Therapeutics, Dental Pathology and Bacteriology, Orthodontia, Oral Surgery, Operative Dentistry, Theoretical, Prosthetic Dentistry, Theoretical, Operative Dentistry, Practical, Prosthetic Dentistry, Practical.

An average of seventy-five per cent. will be required in each subject for Theoretical Examinations, and eighty-five per cent. in the Practical Examinations.

(Signed) John S. Marshall,
*President Examining Board of Dental Surgeons.*

### TRI-STATE DENTAL MEETING.

This will be the largest meeting of the summer, and one of the best. All roads run to Indianapolis, and every dentist in the United States, who is conducting his practice in a legitimate manner, is urged to come and break bread with us. We do not care whether you are a member of a State Association or not if you "do unto others as you would they should do unto you."

The third triennial meeting under the auspices of the State Associations of Ohio, Michigan, and Indiana will occur June 4, 5, and 6, 1901, at Indianapolis, Indiana. The German House, corner of Michigan and New Jersey Streets, has been secured for the meeting and exhibits. The Central Passenger Association has granted a round-trip rate of a fare and a third, on the certificate plan. Any inquiries addressed to 131 East Ohio Street, Indianapolis, will be cheerfully answered.

For further information address,

George E. Hunt,
*Chairman.*

131 East Ohio Street, Indianapolis, Ind.

---

### ILLINOIS STATE DENTAL SOCIETY.

The thirty-seventh annual meeting will be held in Rockford, May 14 to 17, inclusive. All members should make an effort to be present. The Society is always glad to welcome reputable dentists, who are not members, from this and other States.

The local committee has arranged for an informal reception on Tuesday evening in the parlors of the hotel Nelson. A short programme has been prepared, and all in attendance are cordially invited to be present and spend a sociable evening.

C. R. Taylor, Streator, Ill.,
*Chairman Executive Committee.*
J. E. Hinkins, Chicago, Ill.,
*Supervisor of Clinics.*

[The programme of this meeting is quite lengthy, covering sixteen papers and reports, with forty-six clinical operations. It is with regret that space will not permit publication in full.—Ed.]

